# Effects of cocoa consumption on cardiometabolic risk markers: Protocol for a systematic review and meta-analysis of randomized controlled trials

**DOI:** 10.1371/journal.pone.0309824

**Published:** 2024-09-09

**Authors:** Tainah Ortiz Pinto Arisi, Diego Silveira da Silva, Elana Stein, Camila Weschenfelder, Patrícia Caetano de Oliveira, Aline Marcadenti, Alexandre Machado Lehnen, Gustavo Waclawovsky

**Affiliations:** 1 Instituto de Cardiologia do Rio Grande do Sul/Fundação Universitária de Cardiologia, Porto Alegre, Brazil; 2 Hcor Research Institute (IP-Hcor), Hcor, São Paulo, SP, Brazil; 3 Faculdade de Saúde Pública, Universidade de São Paulo (FSP-USP), São Paulo, SP, Brasil; 4 Faculdade Ulbra-Medicina-Gravataí, Gravataí, Brazil; The University of Sydney School of Medicine, AUSTRALIA

## Abstract

**Background:**

Cardiometabolic diseases cover a spectrum of interrelated conditions linked to metabolic dysfunctions and/or cardiovascular disorders, including systemic arterial hypertension, diabetes mellitus, dyslipidemia, and obesity. Cocoa is a rich source of dietary polyphenols and has been associated with cardiovascular health benefits. However, beneficial effects of cocoa consumption and appropriate quantities in decreasing cardiometabolic risk factors have yet to be established. Therefore, we will conduct a systematic review and meta-analysis to examine the effects of cocoa consumption on cardiometabolic risk markers (total cholesterol, HDL, LDL, triglycerides, blood glucose, glycated hemoglobin, waist circumference, abdominal circumference, body mass index, systolic blood pressure and diastolic blood pressure) in adults with or without established cardiovascular risk factors.

**Methods:**

Our review will include all randomized controlled trials published in English, Portuguese and Spanish with no date of publication restrictions evaluating the effects of cocoa consumption on cardiometabolic risk markers selected from the databases MEDLINE (PubMed), LILACS, Cochrane, EMBASE, Web of Science and SciELO, and gray literature. Eligible studies must involve adults (age ≥18y), and the consumption of cocoa or dark chocolate (≥ 70% cocoa), include a control group and evaluate blood pressure, anthropometric measurements, and lipid or glycemic profiles. We will use risk-of-bias 2 (RoB2) tool to assess the risk of bias and the GRADE system to assess the strength of evidence. Statistical analyses will be performed using RStudio for Windows and R package meta.

**Discussion:**

This meta-analysis will summarize existing evidence on the effects of cocoa consumption on cardiometabolic health in adults. Better understanding the effects of cocoa consumption on anthropometric measurements, blood pressure, and lipid and glycemic profiles can provide valuable insights for health professionals to improve dietary recommendations regarding appropriate quantities.

**Trial registration:**

**Systematic Review Registration**: PROSPERO CRD42023484490.

## Background

Cardiometabolic diseases cover a spectrum of interrelated conditions linked to metabolic dysfunctions and/or cardiovascular disorders, including systemic arterial hypertension, diabetes mellitus, dyslipidemias, arteriosclerosis and obesity. Collectively, these conditions are major contributors to morbidity and mortality globally [1], representing a global public health challenge [2,3]. In particular, cardiovascular diseases (CVDs) account for around 17.9 million deaths annually [4], and one-third of them are among adults under the age of 70 [5].

Given the concerning rates of deaths from CVDs, the Framingham risk score is a tool that estimates risk and predicts the 10-year risk of a cardiovascular event based on risk factors, including age, sex, diabetes mellitus, total cholesterol, high-density (HDL) cholesterol, blood pressure (BP), and smoking [6,7]. Interestingly, the INTERHEART Study aimed to identify risk factors associated with myocardial infarction and found similar risk factors to those reported in the Framingham Study despite their different designs and objectives. In fact, obesity [8], inadequate lipid profile [9], diabetes mellitus [10] and arterial hypertension [11] are significantly associated with morbidity and mortality and thus are targets of prevention and treatment strategies. Nutrition plays a major role in the management of these risk markers [12]. One in every five deaths worldwide are caused by conditions associated with unbalanced diets [13,14]. Hence, this evidence supports the importance of evidence-based dietary and nutritional interventions [15].

In addition to macronutrients, foods contain chemical compounds, so-called bioactive compounds, that are associated with health benefits. Polyphenols are bioactive compounds that have been extensively studied in the literature. Studies have demonstrated an association between the consumption of polyphenols and improvements in BP, lipid and glycemic profiles, inflammatory biomarkers, and vascular function, among others [12,16,17].

Nutritional approaches have focused on the potential benefits of bioactive compounds. Cocoa (*Theobroma cacao*) is a fruit rich in polyphenols and has emerged as a dietary agent with potential cardiovascular health benefits. Studies have suggested an association between cocoa consumption and improvements in cardiometabolic risk markers, but there is no consensus in the literature.

Garcia-Yu et al. evaluated regular dietary consumption of 10 g of 99% cocoa dark chocolate (64.5 mg of polyphenols) in 132 women over 6 months and found a favorable decrease in both absolute (–0.63 kg) and relative adiposity (–0.79%), though not sufficient to change body mass index (BMI) levels [18]. In contrast, a meta-analysis of 35 randomized clinical trials (RCTs) that evaluated the effects of cocoa consumption did not report any beneficial effects on body weight and waist circumference [19]. As for lipid profile, a meta-analysis of 10 RCTs demonstrated that cocoa consumption was associated with reduced total cholesterol (–6.23 mg/dL) and LDL levels (–5.90 mg/dL) [20]. However, another study showed that the effects of cocoa consumption on the lipid profile may vary depending on age and intervention duration. Interestingly, the dose-response curve showed no association between total amount of polyphenols consumed and lipid markers [21]. In diabetes mellitus, studies have suggested that cocoa polyphenols may improve insulin signaling and function of pancreatic β-cells [22] and consequently improve glycemic control. Lastly, cocoa also appears to have BP benefits by increasing endothelial nitric oxide synthesis and promoting greater endothelium-dependent vasodilation [23] as well as through inhibition of angiotensin-converting enzyme activity [24,25] resulting in reduced BP levels. This body of evidence suggests that cocoa consumption may have cardiometabolic benefits.

Although several studies have reported promising results of cocoa consumption with positive effects on cardiometabolic risk predictors, despite some uncertainties, there is no consensus on the intervention characteristics (cocoa dosage and frequency, concentration of bioactive compounds, and the form of cocoa) and related effect sizes. Thus, expanding knowledge about this food rich in dietary polyphenols can provide insights to develop nutritional and dietary interventions that are effective in reducing cardiovascular risk.

Considering the consensus in the literature on the major role of risk factors evidenced in several studies, such as the Framingham Heart Study and the INTERHEART Study among others, and that cocoa is easily accessible and its consumption has potential benefits on these risk factors, we will conduct a systematic review and meta-analysis to expand our knowledge and discuss potential effects of cocoa consumption on anthropometric measurements, fasting blood glucose, glycated hemoglobin, lipid profile and BP in adults with or without established cardiovascular risk factors.

## Methods

The study protocol was developed based on PRISMA-P (Preferred Reporting Items for Systematic Review and Meta-Analysis Protocols) 2015 [26] and Cochrane systematic review methodology [27]. The protocol for the systematic review and meta-analysis was registered in the International Prospective Register of Systematic Reviews (PROSPERO) (www.crd.york.ac.uk/PROSPERO/: CRD42023484490, November 30, 2023). The databases used in the meta-analysis will be freely available in the Mendeley Data repository (https://data.mendeley.com/). Any amendments to this protocol will be documented in the main study.

### Eligibility criteria

We used PICOS framework to formulate eligibility criteria as follows: **Population:** age ≥ 18 years, healthy or diagnosed with arterial hypertension, and/or type 2 diabetes mellitus, and/or dyslipidemia, and/or overweight/obesity, and/or myocardial infarction/stroke; **Intervention:** cocoa supplement or chocolate bar (≥ 70% cocoa), cocoa powder, cocoa into snacks; **Comparison:** placebo or < 70% cocoa white/milk chocolate; **Outcomes:** measurements of systolic blood pressure (SBP) and diastolic blood pressure (DBP) (mmHg); BMI (kg/m^2^); body weight (kg); waist or abdominal circumference (cm) as an indicator of central obesity; total cholesterol, HDL cholesterol, LDL cholesterol, triglycerides levels; fasting blood glucose and glycated hemoglobin; **Study:** RCTs only.

This article presents a comprehensive description of the study population of adults with or without established cardiometabolic risk factors (arterial hypertension, and/or type 2 diabetes mellitus, and/or dyslipidemia, and/or overweight/obesity, and/or myocardial infarction/stroke). The inclusion criteria of our review will include studies with two or more arms; an "intervention" arm involving the consumption of cocoa or ≥ 70% cocoa dark chocolate for at least four weeks and a "comparator" arm of placebo or < 70% cocoa white/milk chocolate.

It should be noted that the study populations, methods and intervention will be labeled according to the authors’ description. When information is not available, authors will be contacted by email and asked to respond within 15 days (three attempts). For subgroup analyses in the meta-analysis, only RCTs reporting polyphenol dose for the intervention group and the control group will be selected. The results of subgroup analyses comparing healthy individuals with those with cardiometabolic risk factors will also be discussed.

### Inclusion and exclusion criteria

We will select studies involving adults aged 18 or older and consumption of cocoa (cocoa powder, cocoa into snacks) or ≥ 70% dark chocolate, with no limitation on the amount of cocoa or polyphenols, for at least 4 weeks with detailed assessments of cardiometabolic risk markers, including BP (measured using standard techniques), BMI, waist or abdominal circumference, lipid profile, and glucose metabolism (fasting blood glucose or glycated hemoglobin). Studies combining other dietary interventions with a clearly defined intervention group of cocoa or ≥ 70% dark chocolate intake and a control group will be thoroughly reviewed for inclusion. Studies with participants undergoing medication or diet changes will be eligible if these changes were introduced at least four weeks prior to the start of the intervention or were consistent throughout the study to allow for accurate analysis. Regarding the 4-week intervention, there is no specific guideline or reference, as the outcomes are associated with different timeframes for promoting adaptations. For instance, “polyphenols and weight loss” take 4–6 weeks to show results [28]. Similarly, improvements in “lipid and glycemic profiles with polyphenol supplementation” have been observed in 4–12 weeks, depending on baseline conditions [29,30]. As for changes in blood pressure levels, some studies indicate that cocoa supplementation can reduce blood pressure over a period of 4–8 weeks [23,31]. Therefore, we decided to use 4 weeks as the minimum intervention period.

There will be excluded studies with participants undergoing treatments other than for cardiometabolic conditions; pregnant and post-menopausal women; concomitant use of dietary supplements that are not clearly distinguished from the cocoa intervention; review or protocol studies; animal experimentation studies; and studies of conditions other than those related to cardiometabolic health, such as individuals diagnosed with cancer. Studies involving the same sample published in different journals will be thoroughly reviewed and excluded if duplicate.

### Search strategy

We conducted a prior search in the MEDLINE database via PubMed to ascertain whether the research question of our review meets the FINER (feasible, novel, and relevant) criteria. A search strategy for RCTs was then developed and will be conducted by four independent reviewers (two pairs) in the databases recommended in the Cochrane Handbook for Systematic Reviews of Interventions [27]: MEDLINE, EMBASE (European literature), Web of Science, and CENTRAL–Cochrane Central Register of Controlled Trials (to allow access to trials that are not indexed in MEDLINE and EMBASE). To broaden our search, we will conduct searches of publications from Latin America in LILACS (Latin American and Caribbean Health Sciences Literature/Virtual Health Library [VHL]) and SciELO (Scientific Electronic Library Online). To minimize publication bias, we will also search the gray literature, including OpenGrey and the Brazilian Coordination for the Improvement of Higher Education Personnel (CAPES) Bank of Theses and Dissertations. For unpublished ongoing studies, we will conduct searches in the following clinical trial registries: ClinicalTrial.gov; and Brazilian Clinical Trials Registry (REBEC). Preprint databases will also be included (preprints.org/, biorxiv.org and medrxiv.org). Table 1 details the search terms and search strategies used for each database. We will gather data through careful review of the articles retrieved and the authors will be contacted by email for additional information as needed. Articles in English, Portuguese and Spanish with no date of publication restrictions will be eligible for inclusion. Upon completion of the review, we will undertake an additional search of all databases and registry platforms to ensure the inclusion of the most recent studies.

**Table 1:** Search terms for searches in Medline, EMBASE, Web of Science, CENTRAL–Cochrane Central Register of Controlled Trials, LILACS and SciELO.

**MEDLINE (PubMed)**
(((Chocolate OR Chocolates OR Cocoa OR Cocoa Powders OR Cocoa Powders OR Powder, Cocoa OR Powders, Cocoa OR Cacao OR Cocoa Plant OR Plant, Cocoa OR Theobroma OR Theobroma cacao)) AND ((Blood Pressure OR Pressure, Blood OR Diastolic Pressure OR Pressure, Diastolic OR Systolic Pressure OR Pressure, Systolic OR Pressures, Systolic OR Arterial Pressures OR Pressure, Arterial OR Pressures, Arterial OR Arterial Tension OR Arterial Tensions OR Tension, Arterial OR Tensions, Arterial OR Blood Pressure, Arterial OR Arterial Blood Pressure OR Arterial Blood Pressures OR Blood Pressures, Arterial OR Pressure, Arterial Blood OR Pressures, Arterial Blood OR Mean Arterial Pressures OR Pressure, Mean Arterial OR Pressures, Mean Arterial OR Ambulatory Blood Pressure Monitoring OR Monitoring, Ambulatory Blood Pressure OR Blood Pressure Monitoring Ambulatory Blood Pressure Monitoring OR Body Mass OR Body Weights OR Weight, Body OR Weights, Body OR Index, Body Mass OR Quetelet Index OR Index, Quetelet OR Quetelet’s Index OR Quetelets Index OR Cholesterol OR HDL Cholesterol OR High Density Lipoprotein Cholesterol OR alpha-Lipoprotein Cholesterol OR Cholesterol, alpha-Lipoprotein OR alpha Lipoprotein Cholesterol OR HDL Lipoproteins OR High-Density Lipoprotein OR Lipoprotein, High-Density OR High-Density Lipoproteins OR High Density Lipoproteins OR Lipoproteins, High-Density OR High Density Lipoprotein OR Density Lipoprotein, High OR Lipoprotein, High Density OR Cholesterol, alpha-Lipoprotein OR High Density Lipoprotein Cholesterol OR HDL2 Cholesterol OR HDL3 Cholesterol OR alpha-Lipoprotein Cholesterol OR Low Density Lipoprotein Cholesterol OR beta-Lipoprotein Cholesterol OR Cholesterol, beta-Lipoprotein OR beta Lipoprotein Cholesterol OR LDL Cholesterol OR Cholesteryl Linoleate, LDL OR LDL Cholesteryl Linoleate OR VLDL Cholesterol OR Pre-beta-Lipoprotein Cholesterol OR Cholesterol, Pre-beta-Lipoprotein OR Pre beta Lipoprotein Cholesterol OR Very Low Density Lipoprotein Cholesterol OR Pre beta lipoprotein Cholesterol OR Cholesterol, Pre beta lipoprotein OR Blood Sugar OR Sugar, Blood OR Glucose, Blood OR Hemoglobin, Glycated OR Glycohemoglobin OR Glycohemoglobins OR Glycated Hemoglobins OR Hemoglobins, Glycated OR Hemoglobin, Glycosylated OR Glycosylated Hemoglobin OR Glycated Hemoglobin A1c OR Hemoglobin A1c, Glycated OR Glycosylated Hemoglobin A1c OR Hemoglobin A1c, Glycosylated Hb A1a-2 OR Hemoglobin, Glycated A1a-2 OR A1a-2 Hemoglobin, Glycated OR Glycated A1a-2 Hemoglobin OR Hemoglobin, Glycated A1a 2 OR Glycated Hemoglobin A OR Hemoglobin A, Glycated OR Hb A1a+b OR Hb A1c OR HbA1 OR Glycosylated Hemoglobin A OR Hemoglobin A, Glycosylated OR Hb A1 OR Glycohemoglobin A OR Hemoglobin, Glycosylated A1a-1 OR A1a-1 Hemoglobin, Glycosylated OR Glycosylated A1a-1 Hemoglobin OR Hemoglobin, Glycosylated A1a 1 OR Hb A1a-1 OR Hemoglobin, Glycated A1b OR A1b Hemoglobin, Glycated OR Glycated A1b Hemoglobin OR Hb A1b OR Hemoglobin, Glycosylated A1b OR A1b Hemoglobin, Glycosylated OR Glycosylated A1b Hemoglobin))) AND (Randomized controlled trial[pt] OR controlled clinical trial[pt] OR randomized controlled trials[mh] OR random allocation[mh] OR dORble-blind method[mh] OR single-blind method[mh] OR clinical trial[pt] OR clinical trials[mh] OR "clinical trial"[tw] OR singl*[tw] OR dORbl*[tw] OR trebl*[tw] OR tripl*[tw] OR random*[tw] OR cross-over studies[mh] OR control*[tw] OR volunteer*[tw])
**EMBASE**
chocolate’/exp OR ’cacao’/exp AND ’blood pressure’ OR ’arterial pressure’/exp OR ’blood pressure monitoring’/exp AND ’cholesterol’/exp OR ’low density lipoprotein’/exp OR ’high density lipoprotein’/exp OR ’cholesterol blood level’/exp OR ’very low-density lipoprotein’/exp AND ’hemoglobin A1c’/exp AND ’body mass’/exp AND ’randomized controlled trial’/exp
**CENTRAL–Cochrane Central Register of Controlled Trials**
((Chocolates OR Cocoa Powder OR Powder, Cocoa OR Cocoa Powders OR Powders, Cocoa) AND (Glucose, Blood OR Blood Sugar OR Sugar, Blood OR Hemoglobin A1c, Glycated OR Hemoglobin A1c, Glycosylated OR Glycosylated Hemoglobin A1c OR Glycated Hemoglobin A1c OR Hemoglobin, Glycosylated A1b OR Glycosylated A1b Hemoglobin OR A1b Hemoglobin, Glycated OR A1b Hemoglobin, Glycosylated OR Hb A1bOR Glycated A1b Hemoglobin OR Hemoglobin, Glycated A1b OR A1a-1 Hemoglobin, Glycosylated OR Hemoglobin, Glycosylated A1a-1 OR Hemoglobin, Glycosylated A1a 1 OR Glycosylated A1a-1 Hemoglobin OR Hb A1a-1 OR Hemoglobin, Glycated A1a 2 OR A1a-2 Hemoglobin, Glycated OR Hemoglobin, Glycated A1a-2 OR Hb A1a-2 OR Glycated A1a-2 Hemoglobin OR Glycosylated Hemoglobin OR Hemoglobin, Glycosylated OR Glycated Hemoglobins OR Hemoglobins, Glycated OR Glycated Hemoglobin OR Hemoglobin, Glycated OR Hb A1c OR Hb A1 OR Glycohemoglobin A OR Hemoglobin A(1) OR Hemoglobin A, Glycated OR Glycosylated Hemoglobin A OR Hemoglobin A, Glycosylated OR Hb A1a+b OR HbA1 OR Blood Pressure OR Pressure, Blood OR Diastolic Pressure OR Pressure, Diastolic OR Pulse Pressure OR Pressure, Pulse OR Systolic Pressure OR Pressures, Systolic OR Pressure, Systolic OR Blood Pressure, Arterial OR Tensions, Arterial OR Arterial Blood Pressures OR Blood Pressures, Arterial OR Pressures, Arterial Blood OR Arterial Blood Pressure OR Pressure, Arterial Blood OR Arterial Pressures OR Arterial Tensions OR Pressure, Arterial OR Pressures, Arterial OR Arterial Tension OR Tension, Arterial OR Pressure, Mean Arterial OR Arterial Pressures, Mean OR Arterial Pressure, Mean OR Pressures, Mean Arterial OR Mean Arterial Pressure OR Mean Arterial Pressures OR Blood Pressure Monitoring, Home OR Home Blood Pressure Monitoring OR Ambulatory Blood Pressure Monitoring OR Monitoring, Ambulatory Blood Pressure OR Self Blood Pressure Monitoring OR Blood Pressure Monitoring, Self OR Body Weight OR Body Weights OR Weight, Body OR Weights, Body OR Body Weight Change OR Change, Body Weight OR Changes, Body Weight OR Body Weight Maintenance OR Maintenances, Body Weight OR Maintenance, Body Weight OR Body Weight Maintenances OR Ideal Body Weight OR Ideal Body Weight Formula OR Normal Body Weight OR Body Weight, Normal OR Body Weights, Normal OR Body Weights, Ideal OR Ideal Body Weights OR Body Weight, Ideal OR Normal Body Weights OR Ideal Body Weight Chart OR Body-Weight Trajectories OR Trajectories, Body-Weight OR Body Weight Trajectory OR Trajectory, Body-Weight OR Body Mass Index OR Quetelet’s Index OR Quetelet Index OR Index, Quetelet OR Quetelets Index OR Index, Body Mass OR Blood Cholesterol OR High Density Lipoprotein Cholesterol OR Cholesterol, alpha-Lipoprotein OR alpha Lipoprotein Cholesterol OR alpha-Lipoprotein Cholesterol OR HDL(2) Cholesterol OR HDL(3) Cholesterol OR HDL Cholesterol OR HDL2 Cholesterol OR Cholesterol, HDL2 OR Cholesterol, HDL3 OR HDL3 Cholesterol OR Cholesterol, LDL OR Low Density Lipoprotein Cholesterol OR beta Lipoprotein Cholesterol OR beta-Lipoprotein Cholesterol OR LDL Cholesterol OR Cholesterol, beta-Lipoprotein OR LDL Cholesteryl Linoleate OR Cholesteryl Linoleate OR Very Low Density Lipoprotein Cholesterol OR Cholesterol, Pre-beta-Lipoprotein OR Pre-beta-Lipoprotein Cholesterol OR Pre beta Lipoprotein Cholesterol OR Cholesterol, Pre beta lipoprotein OR Pre beta lipoprotein Cholesterol OR VLDL Cholesterol))
**LILACS**
English: (Cocoa OR Cocoa Plant OR Plant, Cocoa OR Theobroma OR Theobroma cacao OR Chocolates OR Chocolate OR Cocoa Powder OR Cocoa Powders OR Powder, Cocoa OR Powders, Cocoa) AND (Blood Pressure OR systolic OR diastolic OR BMI OR Body Weights OR Body Mass Index OR Blood Glucose OR Glycated Hemoglobin OR Cholesterol OR HDL OR LDL OR VLDL)) Portuguese: (Cacau OR Cacau Planta OR Planta, Cacau OR Theobroma OR Theobroma cacao OR Chocolates OR Chocolate OR Cacau em Pó OR Cacau em Pó OR Pó, Cacau OR Pós, Cacau) and (pressão arterial OR sistólica OR diastólica OR IMC OR peso corporal OR índice de massa corporal OR glicose OR hemoglobina glicada OR colesterol OR HDL OR LDL OR VLDL) Spanish: ((Cacao OR Planta de cacao OR Planta, Cacao OR Theobroma OR Theobroma cacao OR Chocolates OR Chocolate OR Cacao en polvo OR Cacao en polvo OR Polvo, Cacao OR Polvos, Cacao) and (Presión arterial OR sistólica OR diastólica OR IMC OR peso corporal OR Pesos corporales OR índice de massa corporal OR Glucosa OR hemoglobina glucosilada OR colesterol OR HDL OR LDL OR VLDL))
**WEB OF SCIENCE**
(Chocolate OR Chocolates OR Cocoa OR Cocoa Powders OR Cocoa Powders OR Powder, Cocoa OR Powders, Cocoa OR Cacao OR Cocoa Plant OR Theobroma) AND (Blood Pressure OR Body Mass OR Body Weights OR Cholesterol OR Cholesterol Total OR HDL Cholesterol OR High Density Lipoprotein Cholesterol OR LDL Cholesterol OR Blood Sugar OR Glucose, Blood OR Glycated Hemoglobin A1c) AND (Randomized controlled trial OR controlled clinical trial OR clinical trial OR random OR cross-over studies)
**SciELO (Advanced Searches)**
(Cocoa OR Chocolates OR Chocolate) AND (Blood Glucose) OR (Glycated Hemoglobin) OR (Blood Pressure) OR (Body Weights) OR (Body Mass Index) OR (Cholesterol)

The main search terms will include “cocoa,” “Theobroma cacao,” and “dark chocolate” (Table 1). To increase accuracy and sensitivity of our searches, the search terms for the study design (RCT) will be entered in the databases MEDLINE [32] and EMBASE [33] (Table 1). Four reviewers will independently select studies after an initial screening of titles and abstracts. When there will be no sufficient information in the abstract, the reviewers will read the full text of the article. Any disagreements between the reviewers will be resolved through discussion and, if no consensus can be reached, a fifth reviewer (WG) will be consulted.

### Data extraction and management

After completing our searches in each database, all articles retrieved will be exported as “.ris” or “.enib” files and imported into Rayyan reference manager [34]. Duplicate studies will be removed using the deduplication function. Our blinded reviewers will manually check for remaining duplicates by reviewing titles, authors, year of publication and abstracts.

Our reviewers will independently screen eligible articles based on their titles and abstracts. They will use Rayyan application “to include” articles that meet the eligibility criteria. Those that do not meet the inclusion criteria will be marked as “excluded” and categorized by reason for exclusion (i.e., ineligible outcomes or population and non-RCT design). When it is not clear whether a study should be included, it will be marked as “undecided”. In the last step, our reviewers will compare the articles screened for any discrepancies. If there is not enough information in the abstract, we will retrieve and read the full-text article. Disagreements will be resolved by consensus and any disagreements on inclusion criteria will be resolved by a fifth reviewer (WG). Cohen’s kappa statistic will be used to measure the agreement between the reviewers.

The four blinded reviewers will independently conduct data extraction. When studies will be considered relevant and selected, the main data will be extracted and compiled in a pre-structured Excel 2019 database divided into columns: reviewer; author; year; journal; country; inclusion and exclusion criteria; analysis strategy; number of individuals included and excluded; number of individuals analyzed; sex; age; follow-up period; nutritional supplement taken; how it was supplemented; daily dose; and risk of bias. For the main analysis, baseline and post-intervention data will be extracted (mean and dispersion measures) for the following markers: BP, body weight, BMI, waist and abdominal circumference, blood glucose, glycated hemoglobin, total cholesterol, HDL cholesterol, LDL cholesterol and triglycerides. For the extraction of data from eligible studies with results presented in graphs, we will contact the authors by email to obtain these data or use GetDate Graph Digitizer 2.26 to extract the data.

### Risk of bias

The risk of bias of RCTs will be assessed using Cochrane risk of bias (RoB) 2 tool included in the Cochrane Handbook [27,35,36]. The assessment is based on a set of five domains of bias rated as low, high, or unclear risk of bias: bias arising from the randomization process; bias due to deviations from intended interventions; bias due to missing outcome data; bias in measurement of the outcome; bias in selection of the reported result. When participants are not blinded, studies will be classified as high risk of bias in the “blinding of participants and personnel” domain. No study will be excluded based on the risk of bias assessment. The risk of bias will be analyzed for the primary outcomes of interest in our review.

### Risk of overall bias in systematic reviews

We will assess the certain of evidence using the Grading of Recommendations Assessment, Development and Evaluation (GRADE) tool [37,38]. This tool assesses confidence in paired effect estimates and classifies treatment in a meta-analysis (high, moderate, low and very low) in the following domains [37,38]: study design, methodological limitations (risk of bias), inconsistency, indirectness of evidence, imprecision, publication bias, magnitude of effect, dose-response gradient, and residual confounders.

### Analysis strategy

We will carry out statistical analyses to estimate the effects of cocoa and/or ≥ 70% cocoa dark chocolate consumption on anthropometric measurements, BP, and lipid and glycemic profiles compared to a control group. For outcomes with the same unit, summary effect estimates will be expressed as mean difference (MD) and related 95% confidence interval (95% CI). For outcomes with different units, summary effect estimates will be expressed as standardized mean difference (SMD) and related 95% CI. The SMD expresses the size of the intervention effect in each study relative to the variability in outcome measurements observed in that study. Given that the SMD provides an overall interpretation of the results (absolute unit), we will convert SMD into units of the most common measurement instrument or into proportions (%) so that the data are presented in a language more adequate to the outcome analyzed.

In studies involving humans, inconsistencies across studies are to be expected due to different characteristics of the participants (clinical heterogeneity) or methods (methodological heterogeneity), with different effect sizes between studies. Therefore, to incorporate variability in the meta-analysis and to summarize the data, MDs or SMDs from individual studies will be pooled using a random-effects model. Therefore, we will use a random-effects model to incorporate variability in the meta-analysis. Since the 95% CI from random effects refer to uncertainty in the location of the mean effects across studies, we will consider the calculated values for a 95% prediction interval (95% PI) as they reflect the interval of uncertainty of the effects to be expected in future RCTs [39]. To assess the consistency of cocoa and/or ≥ 70% cocoa dark chocolate effects across studies, the degree of heterogeneity will be tested using the inconsistency test by Higgins (*I*^*2*^) for every pairwise comparison [40,41]. To explore heterogeneity, we will conduct subgroup analyses and meta-regression analyses (≥ 10 studies) for effect modifiers with normal distribution in a quartile-quartile plot (*qq-plot*) and confirm it using the Shapiro-Wilk test (p > 0.05) [42]. Non-normal data will be normalized before performing meta-regression analysis. To remove discrepant data from the meta-analysis, forest plots will be constructed to display the effect estimates across studies and detect potential outliers based on non-CI overlapping that is due to heterogeneity [41]. Potential effect modifiers (e.g., age, BMI, number of interventions and total polyphenol content) will be analyzed separately. If there will be significant heterogeneity that cannot be explained, we will not perform a meta-analysis, but we will present individual intervention effect estimates from the studies. Considering that selective publication and/or suppression of specific results cause bias and consequently affect the validity of the results [43], if applicable (≥ 10 studies; more than one study with significant statistical data; studies with different sample sizes), we will perform the Egger’s test using a funnel plot to assess potential publication bias in the meta-analysis [44,45]. If publication bias is detected (Egger’s test, p < 0.1), we will use the trim-and-fill method to identify and correct for funnel plot asymmetry by adding information of the bias-corrected data to the original data (prior to trimming and imputation of data by the trim-and-fill method) [46,47]. Alternative analyses to the primary analysis of data including sensitivity analysis will be performed to assess the robustness of our decisions (e.g., *imputation method used* to *impute* missing values; inclusion of studies with high risk of bias, data from conference abstracts etc.) [48]. Dispersion measures expressed as confidence intervals (CI) or standard errors (SE) will be converted to standard deviation (SD = EP * √n) before the analysis. For eligible studies that do not report SD of differences, it will be estimated using an imputed correlation coefficient (CC) of 0.5 [49]. CC equation (section 6.5.2.8, Cochrane Handbook): Δ SD = √ SD^2^ baseline + SD^2^ final–(2 * CC * SD baseline * SD final). Two-tailed tests will be used at a significance level of p< 0.05. Data modeling will be performed with RStudio (version 1.3.959) using the R package meta (version 3.6.1) for Windows.

## Discussion and conclusions

Cocoa is recognized for its abundance of flavonoids, compounds known for their antioxidant and anti-inflammatory properties. Flavonoids, especially flavanols found in cocoa, have been associated with positive cardiovascular effects, especially on lipid profiles [21,22], fasting blood glucose [50,51], and BP [24,25]. Furthermore, studies indicate that flavonoids can reduce oxidized LDL cholesterol and improve lipid profiles, which are crucial for preventing cardiovascular diseases [17,21]. Additionally, cocoa can have an effect on fasting blood glucose, which suggests potential benefits for individuals with insulin resistance or type 2 diabetes [42,43]. Proposed mechanisms include improved insulin sensitivity and reduced oxidative stress that contribute to a healthier metabolic environment.

The accumulated evidence has shown that cocoa is a promising functional food for cardiometabolic health and supports ongoing research into its effects and potential therapeutic applications. These findings suggest that cocoa, which is a rich source of flavonoids and other bioactive compounds, would be a cardioprotective substance. However, there is still uncertainty regarding its ideal dosage, concentration of bioactive compounds to promote cardioprotective benefits, frequency and form of consumption, as well as the magnitude of improvement in individual CVD risk factors.

In conclusion, understanding the potential effects of cocoa consumption is crucial as it may offer valuable insights to develop dietary strategies for managing cardiometabolic risk factors across diverse populations. A meta-analysis with different samples, including individuals with arterial hypertension, type 2 diabetes mellitus, dyslipidemia, and/or overweight/obesity, can provide evidence to support previous findings and deepen our understanding of effective cocoa doses and forms of consumption. These insights could help refine current dietary recommendations and develop evidence-based guidelines for a heart-healthy diet including cocoa. The systematic review and meta-analysis is nearing completion, with publication bias and strength of evidence being analyzed as the last methodological step.

## Supporting information

S1 Checklist PRISMA-P (Preferred Reporting Items for Systematic review and Meta-Analysis Protocols) 2015 checklist: Recommended items to address in a systematic review protocol. (PDF)

## Abbreviations

SBP: systolic blood pressure
DBP: diastolic blood pressure
RCTs: randomized controlled trials
PRISMA-P: Preferred Reporting Items for Systematic Review and Meta-Analysis Protocols
PICOS: Population, Intervention, Comparison, Outcome, and Study
BMI: body mass index
HDL: high-density lipoprotein
LDL: low-density lipoprotein
